# Investigating the Existence of Ribosomal Protein L5 Gene in Syrian Strain of *Leishmania tropica* Genome: Sequencing It and Evaluating Its Immune Response as DNA Vaccine

**DOI:** 10.1155/2021/6617270

**Published:** 2021-05-20

**Authors:** Mohammad Maarouf, Alyaa A. Abdlwahab

**Affiliations:** ^1^Department of Microbiology, Faculty of Pharmacy, Damascus University, Syria; ^2^Department of Biochemistry, Faculty of Pharmacy, Damascus University, Syria

## Abstract

Cutaneous leishmaniasis in Syria is caused mainly by *Leishmania tropica*. It represents a serious health problem, which has aggravated further after the civil war in the country. Until now, there are no effective protective strategies, safe therapy, or efficacious vaccine to protect from this infection. DNA vaccines represent a promising approach for achieving protection against leishmaniasis. The L5 ribosomal protein plays fundamental roles in the assembly process of the ribosome subunits, so this study has chosen the ribosomal protein L5 gene to design a DNA vaccine against *Leishmania tropica* infection. After proving the existence of the ribosomal protein L5 gene in a Syrian strain of *Leishmania tropica* (LCED Syrian 01), it was sequenced and cloned into a pCI plasmid, and the designed DNA vaccine was administered to BALB/c mice. The protective response was evaluated by measuring lesion development in immunized BALB/c mice for 6 weeks after challenging mice with the parasite. RT-qPCR was used to quantify IL-12, IFN-*γ*, and IL-4 in draining lymph nodes (DLNs) of immunized mice. In the final week, the parasite burden was determined in footpad lesions and local draining lymph nodes (DLNs). This study demonstrated the presence and expression of the ribosomal protein L5 gene in the Syrian strain of *Leishmania tropica* promastigotes. The sequence of the ribosomal protein cDNA L5 gene was determined and published in Genbank. The gene size was 918 bp. Expression was also demonstrated at the level of cDNA. This study also demonstrated that vaccination with the ribosomal protein L5 gene induces TH1 response in immunized mice. This response prevents the partial development of a skin lesion of *Leishmania*.

## 1. Introduction

Leishmaniasis is a neglected tropical disease (NTD) that is caused by the obligate protozoan parasite *Leishmania* sp. which is transmitted by the bite of infected female sand flies. The three main clinical forms of leishmaniasis are cutaneous, visceral, and mucocutaneous as discussed by Sunyoto et al. [1]. Cutaneous leishmaniasis CL is the most common and prevalent form of leishmaniasis with 90% of all cases occurring in seven countries (as discussed by Hepburn [2]): Afghanistan, Algeria, Brazil, Iran, Peru, Saudi Arabia, and Syria. In Syria, 90% of the reported CL cases are caused by *Leishmania tropica* as discussed by Haddad et al. [3].

Treatment of cutaneous leishmaniasis is currently based on therapeutic chemicals with serious limitations like high cost, toxicity, lack of efficacy, and drug resistance, so vaccination remains the best hope for controlling all forms of leishmaniasis. One of the most critical global public-health priorities is the development of a safe, effective, and affordable antileishmanial vaccine as discussed by Seyed et al. [4].

DNA vaccines represent a promising approach for vaccination against diseases in which TH1 responses and cell-mediated immunity are required, such as with leishmaniasis. This approach of immunization elicits both humoral and cellular immune response against the encoded antigen as discussed by Tahereh and Rafati [5].

In leishmaniasis, resistance is associated with the production of IL-12 by antigen-presenting cells (APC), macrophages, and dendritic cells that results in differentiation and proliferation of the TH1-subset (CD4+ T cells) that produces IFN-*γ*. This will ultimately lead to the activation of parasite-infected macrophages that will kill the intracellular parasites by nitric oxide (NO) as discussed by Stenger et al. [6]. Production of anti-inflammatory cytokines like IL-4, IL-10, IL-13, and TGF-*β* contributes to the failure of controlling the infection as discussed by Alexander and Bryson [7].

In this study, we have decided to evaluate the immune response of the DNA vaccine encoding the ribosomal protein L5 gene of *Leishmania tropica*due to the widespread occurrence of *Leishmania tropica* in the country. We chose the ribosomal protein L5 gene to design the needed vaccine which prevents leishmaniasis infection, because the ribosomal protein L5 has fundamental roles in the life of a parasite. Among the most conserved protein families are the ribosomal proteins (e.g., ribosomal protein L5). Ribosomal proteins have specific and strong immunogenic features and are generally designated as pan antigens. These pan antigens can provide the immunomodulatory properties needed for vaccine design as discussed by Requena et al. [8]and Santarém et al. [9].

Ribosomal proteins had elicited a protective response when administered as DNA vaccines in many bacteria and parasites, such as ribosomal protein L9 in *Brucella abortus*, as discussed by Jain et al. [10]; ribosomal protein L7/L12 in *Brucella abortus*, as discussed by Kurar and Splitter [11]; and ribosomal protein S4 in *Schistosoma japonicum* as discussed by Ping et al. [12]. Recombinant ribosomal proteins L3 or L5 with the TH1-inducing adjuvant had elicited protective responses against CL caused by *L. major* as discussed by Ramírez et al. [13].

The ribosomal protein L5 gene has amastigotic and promastigotic expression as discussed by Biyani et al. [14]; this protein ensures the success of the assembly process of the ribosome subunits and thus has a fundamental role in the protein synthesis process as discussed by Matthew and Dreyfuss [15].

This study is aimed at detecting the existence of the ribosomal protein L5 gene in the genome of the LCED Syrian 01 *Leishmania* strain to prove the expression of this gene in these parasites, clone it into an expression plasmid, and evaluate the efficacy of the designed DNA vaccine (pCI-L5) against infection with the LCED Syrian 01 *Leishmania* strain. BALB/c mice were immunized intramuscularly in the left posterior thigh muscle three times at an interval of two weeks with the designed vaccine. BALB/c mice were challenged in the right footpad with 10^6^ promastigotes of *L. tropica* 2 weeks after the last immunization.

Our results show that the pCI-L5 vaccine induces partial protection against infection, decreases the parasite burden in a footpad lesion and local lymph nodes, induces a higher gene expression of IL-12 and IFN-*γ*, and induces a lower gene expression of IL-4 in the draining lymph nodes of vaccinated mice in comparison with the control group, which indicate the ability of the designed vaccine to induce a TH1 response that prevents the partial development of a skin lesion of *Leishmania*.

## 2. Materials and Methods

### 2.1. Primer Design for Polymerase Chain Reaction PCR

An alignment for the sequence of the ribosomal protein L5 gene in other *Leishmania* species was made because the sequence of the gene is not known in *L. tropica*. The CLC Free Workbench 7 program, Vector NTI Express programs, and EMBOSS_needle tools were used to make this alignment. Primers were designed using http://www.idtdna.com/calc/analyzer. This site was used to determine melting temperatures and the possibility of confirmation of self-dimers, hairpins, and heterodimers. http://www.geneinfinity.org/sms/sms_primanalysis.html tool was used to determine if the restriction enzymes can cut the gene. High purified primers were ordered from Alpha DNA, Montreal, Canada as discussed by Orabi et al. [16]. Final sequences for primers are as follows:

- Forward: 5′-AGA ATT C AT GCC GTT CGT CAA GGT CGT G-3′
- Reverse: 5′-GT T CTA GA T TAC TTG CCG AGG CGC TC-3′

EcoR1 and XbaI restriction sites included for direct cloning in the pCI mammalian expression vector are underlined which promotes constitutive expression of the cloned DNA inserts in mammalian cells as discussed by Orabi et al. [16].

### 2.2. Parasite Culture and Genomic DNA Extraction

Syrian strain *Leishmania tropica* promastigotes (LCEBS, Damascus, Syria) were cultured in a RPMI-1640 medium (Lonza, Switzerland) and supplemented with 5% FCS (Fetal Calf Serum; Cytogen, GmbH, Germany). Genomic DNA was extracted from these promastigotes by a DNA extraction kit (Thermo Fisher Scientific, Lithuania) according to the manufacturer's instructions.

### 2.3. Total RNA Isolation and cDNA Synthesis

A GeneJET RNA Purification Kit (Thermo Fisher Scientific, USA) was used to extract total RNA from harvested *Leishmania tropica* promastigotes. mRNA was reverse transcribed into single-stranded cDNA using the RevertAid First Strand cDNA Synthesis Kit (Thermo Fisher Scientific, Lithuania) using oligo dT primers according to the manufacturer's instructions.

### 2.4. PCR Amplification of Ribosomal Protein L5

A thermal cycler (Bio-Rad, USA) was used to amplify DNA and cDNA of the ribosomal protein L5 gene using a PCR master mix (Thermo Fisher Scientific, Lithuania) and the primers mentioned above. Gradient annealing temperatures were used to optimize PCR protocol. Reactions were performed in a 25 *μ*l mixture containing 1 *μ*l from each primer at a final concentration of 0.4 *μ*M in a final volume of 25 *μ*l or 2.5 *μ*l template DNA (250 ng) or 2.5 *μ*l cDNA (240 ng), 12.5 *μ*l PCR Master Mix, and 8 *μ*l PCR water. Thermal cycling conditions were 95°C for 5 min followed by 35 cycles of 95°C for 1 min, 59°C for 45 sec, 72°C for 1 min, and finally 72°C for 5 min.

### 2.5. Plasmid Construction and Purification

The GeneJET PCR Purification Kit (Thermo Fisher Scientific, Lithuania) was used to purify the PCR product containing the ribosomal protein L5 coding sequence, as per the manufacturer's instructions. Double digest with EcoR1 plus XbaI was applied on purified L5-cDNA and the pCI mammalian expression vector (Promega, United States). T4 DNA ligase (Thermo Fisher Scientific, Lithuania) was used to ligate digested L5-cDNA with the pCI mammalian expression vector. pCI-L5 clones were transformed into *E. coli* TOP10. Recombinant plasmids were extracted from transformed *E. coli* TOP10 by alkaline sodium dodecyl sulphate (SDS) lysis and a column purified of endotoxins (GeneJET Plasmid Maxiprep Kit; Thermo Fisher Scientific, Lithuania) according to the manufacturer's instructions.

### 2.6. Mice Vaccination

Mice were purchased from the Scientific Research Center, Damascus, Syria. Mice were divided into two groups, the study group and the control group, with each group containing 25 female BALB/c mice (4-6 months old). Mice of the study group were immunized three times at 2-week intervals by the administration of 100 *μ*g of pCI-L5 vaccine suspended in 100 *μ*l of phosphate saline buffer (PBS) intramuscularly in the left posterior thigh muscle. Mice of the control group were inoculated three times at 2-week intervals with empty plasmid (pCI).

### 2.7. Parasite Challenge

Two weeks after the last immunization, the control and study groups were challenged by subcutaneous inoculation with 10^6^ stationary-phase promastigotes of *Leishmania tropica* (LCED Syrian 01) in the right foot pad. A metric calliper (Mitutoyo, Kawasaki Kanagawa, Japan) was used to measure footpad thicknesses, calculated as the thickness of the left footpad minus the thickness of the right footpad. The comparison was done between the vaccinated mice group and the control group.

### 2.8. Estimation of Parasite Load

The limiting dilution assay method was used to estimate the parasite load six weeks postchallenge as discussed by Fatemeh et al. [17]. Previously, three mice were sacrificed from each group. The feet were disinfected with 70% ethanol, and the draining lymph nodes near the area of the lesion were taken. A single-cell suspension of draining lymph nodes was made and resuspended in 10 ml biphasic medium (C-M199; Thermo Fisher Scientific, Lithuania). The surface of the infected footpad was disinfected, and tissue was removed and placed in a 1.5 ml tube containing 0.3 ml biphasic medium (C-M199; Thermo Fisher Scientific, Lithuania). Then, the tissue was disrupted manually. The lymph node cell and lesion cell suspensions were serially diluted (tenfold) in culture medium (RPMI-1640; Lonza, Switzerland) and added to 96-well plates (CELLSTAR® Cell Culture Microplates, Germany). The plates were coated with paraffin papers and placed in an incubator at 25°C for 7 days. The highest dilution, at which parasites can be seen by microscopic inspection, was recorded as parasite load in the site of inoculation and in draining lymph nodes (DLNs) of BALB/c mice.

### 2.9. Real-Time RT-qPCR for Cytokine Assay

qPCR was used to determine the quantity of IL-12, IFN-*γ*, and IL-4 in weeks 2, 4, and 6 after challenge, in order to monitor the cytokine gene expression in draining lymph nodes (DLNs); three mice were sacrificed at each time point. Scalpels were used to remove the lymph nodes to avoid contamination, and the lymph nodes were hashed with Potter grinders, homogenized well in 1.5 ml microtubes, and then filtered. The GeneJET RNA Purification Extraction Kit was used to extract RNA from draining lymph nodes (DLNs) from every mice group. An RNase-free DNase Set was used to remove any trace of genomic DNA. Reverse transcriptase (200 U) was used to reverse transcribe RNA (2 *μ*g). Real-time PCR was done using StepOne Real-Time PCR system (Applied Biosystems) that uses SYBR Green (SYBR Green Master Mix), and 200 ng of cDNA was used as a template (cDNA Omniscript RT kit; Qiagen). The forward and reverse primers for cytokine genes as discussed by Overbergh et al. [18] and HPRT genes as discussed by Liu et al. [19] are as follows:

- IL-4-RV 5′-GAAGCCCTACAGACGAGCAGCTCA-3′
- IL-4-FW 5′-ACAGGAGAAGGGACGCCAT-3′
- IFN-*γ*-RV 5′-TGGCTCTGCAGGATTTTCATG-3′
- IFN-*γ*-FW 5′-TCAAGTGGCATAGATGTGGAAGA A-3′
- IL-12 -RV 5′-AACTTGAGGGAGAAGTAGGAATGG-3′
- IL-12-FW 5′-GGAAGCACGGCAGCAGAATA-3′
- HPRT-RV 5′-CCAGCAAGCTTGCAACCTTAACC A-3′
- HPRT-FW 5′-GTAATGATCAGTCAACGGGGGAC-3′

The hypoxanthine phosphoribosyltransferase (HPRT) gene was used to normalize the mRNA expression levels of this gene, and this expression was calculated as the difference by *n*-fold of the expression in activated cells compared with its naïve parallel. Reactions were performed in 25 *μ*l of a mixture containing 1 *μ*l from each primer at a final concentration of 0.4 *μ*M in a final volume of 25 *μ*l, 2.5 *μ*l of template cDNA (200 ng), 12.5 *μ*l of SYBR Green Reaction Master Mix (Applied Biosystems, USA), and 8 *μ*l of PCR water. PCR conditions are as follows: an initial denaturation step at 95°C for 2 min followed by 35 cycles of denaturation at 95°C for 1 min and annealing/extension at 61°C for 1 min. The Mx3005P (Agilent Technologies, USA) was used to process and analyze reactions. Relative quantitation was used to calculate gene expression using the comparative Ct method (ΔΔCt), as previously described, and 0.02 was determined as threshold. Fold change (2^−ΔΔCt^) was used to express the gene expression, in relation to samples from the control group that were used as calibrators.

### 2.10. Statistical Analysis

The values expressed by average *X* and SD were compared by the mixed ANOVA. The results were considered statistically significant when *P* < 0.05.

## 3. Results

### 3.1. Evaluation of Genomic DNA and Total RNA Extraction Quality and Purity

**Figure 1:**
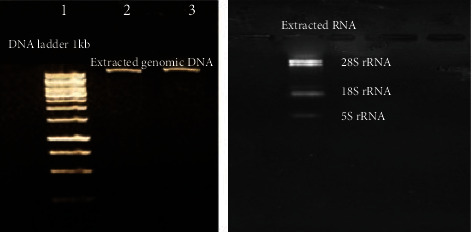
(a) Electrophoresis of extracted *L. tropica* genomic DNA. Lane 1: DNA ladder 1 kb. Lanes 2 and 3: extracted *L. tropica* genomic DNA. (b) Electrophoresis of extracted *L. tropica* total RNA.

Extracted DNA electrophoresis on a 1% agarose gel showed only one band (Figure 1(a)) which demonstrated that degradation of the extracted DNA did not happen. The purity of the DNA was 1.78, which showed a high degree of purity in the extracted DNA. The purity of the extracted RNA was 2.3, which reveals good purity of the extracted RNA. The electrophoresis of the extracted RNA on a 1.5% agarose gel (Figure 1(b)) showed a standard profile, and there was no contamination with genomic DNA.

### 3.2. Polymerase Chain Reaction

**Figure 2:**
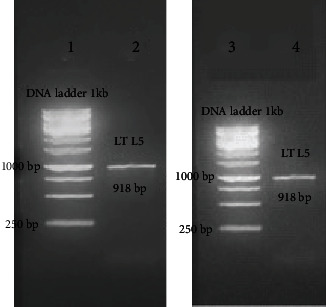
Amplification products of *L. tropica* ribosomal protein L5 on 1% agarose gel electrophoresis stained with ethidium bromide. Lanes 1 and 3: DNA ladder 1 kb. Lane 2: ribosomal protein L5-DNA amplification product approximately 918 bp. Lane 4: ribosomal protein. L5-cDNA amplification product approximately 918 bp.

PCR protocol was optimized using gradient annealing temperatures, and the most suitable annealing temperature was 59°C. Gel electrophoresis of PCR products on the level of DNA and cDNA (Figure 2) showed only one band, and its size was approximately 918 base pairs.

### 3.3. Cloning and Sequencing of the Ribosomal Protein L5 cDNA

**Figure 3:**
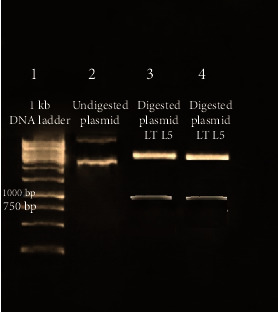
1% agarose gel electrophoresis stained with ethidium bromide of the extracted, double-digested plasmid. Lane 1: DNA ladder 1 kb. Lane 2: undigested plasmid band. Lanes 3 and 4: digested plasmid shows a band of the ribosomal protein L5 gene and a band of the plasmid.

**Figure 4:**
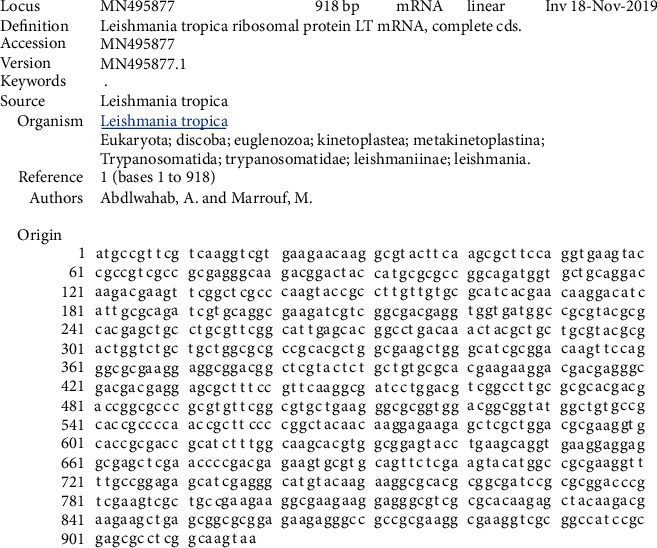
Sequencing of the ribosomal protein L5 gene in the Syrian strain of *L. tropica*.

The results of the PCR products of 5 colonies have shown that three of them were positive and showed the band of the ribosomal protein L5 gene. Plasmids were extracted from positive colonies, purified and double digested, and the electrophoresis of the digested plasmid showed two bands: one of the digested plasmid and the other of the ribosomal protein L5 gene (Figure 3). The ribosomal protein L5 cDNA clone was identified and sequenced. The sequence was submitted to Genbank under accession number MN495877.1 (Figure 4). Ribosomal protein L5 containing 305 amino acids was predicted by the deduced amino acid sequence. The Supplementary Material (available here) shows the complete nucleotide sequence of the 918 bp cDNA insert and the comparison of the deduced amino acid sequence with other species. The Geneious Prime program was used to compare this predicted sequence with the sequences of proteins coded by cDNA of ribosomal protein L5 in other species of *Leishmania* such as *L. major*, accession No. XM_003722531.1; *L. mexicana*, accession No. XM_003879135; *L. donovani*, accession No. XM_003864722.1; and *L. infantum*, accession No. XM_003392749.1. Interestingly, the *L. tropica* ribosomal protein L5 gene shows significant similarities with the ribosomal protein L5 gene of *L. infantum* (99.46%), *L. major* (98.58%), *L. donovani* (99.46%), and *L. mexicana* (99.67%) using the CLC Main Workbench.

### 3.4. The Effect of Mice Vaccination on Infection Development and Parasite Load

**Figure 5:**
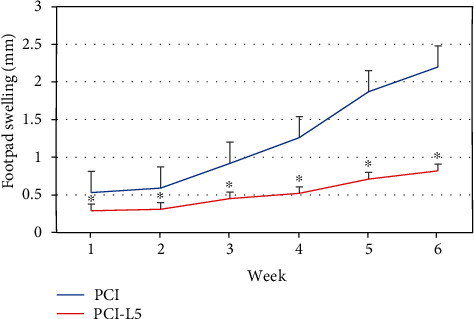
Footpad swelling in BALB/c mice immunized intramuscularly in the left posterior thigh muscle 3 times at 2-week intervals, with 100 *μ*g of empty plasmid as the control group pCI and 100 *μ*g of pCI-L5 vaccine as the study group pCI-L5. After challenge in the right footpad with 10^6^*Leishmania tropica* promastigotes 2 weeks after the last immunization. As geometric mean ± SD. ^∗^*P* < 0.05, significant increase in footpad swelling in the vaccinated group compared to the control group.

The footpad swelling in mice vaccinated with the pCI-L5 vaccine was compared with that of the control. A 62.72% decrease was observed in the vaccinated group compared with the control group after 6 weeks from the last vaccination (Figure 5). This reduction was statistically significant with *P* < 0.05.

**Figure 6:**
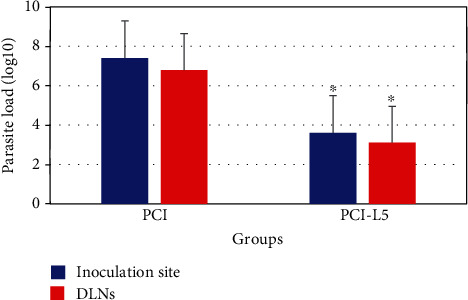
Log 10 of parasite burden in the site of inoculation and lymph nodes six week after the challenge. Parasite load was determined by limiting dilution after S.C. inoculation of 10^6^*Leishmania tropica* promastigotes into the right footpad. As geometric mean ± SD. ^∗^*P* < 0.05, significant decrease in the site of inoculation and lymph nodes parasite burden in the vaccinated group compared to the control group.

The parasite load was 7 × 10^3^ in the inoculation site and 5 × 10^3^ in the draining lymph nodes of the vaccinated group pCI-L5 (Figure 6). Compared to the control group, the parasite burden was reduced by 3.8 log and 3.1 log in the lesion and lymph nodes of the vaccinated group, respectively. These reductions of parasite burden in the lesion and lymph nodes were statistically significant with *P* < 0.05.

### 3.5. IFN-*γ*, IL-12, IL-4 Gene Expression Analysis by RT-qPCR

**Figure 7:**
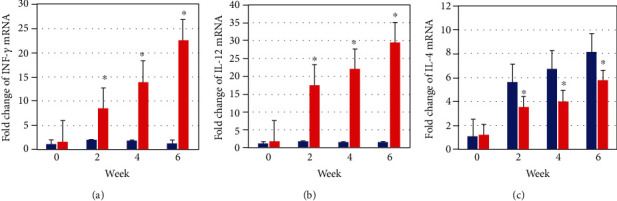
(a) IFN-*γ*, (b) IL-12, and (c) IL-4 gene expression in the draining lymph nodes obtained from BALB/c mice inoculated with 100 *μ*g pCI-L5 vaccine. The messenger RNA (mRNA) for IFN-*γ*, IL-12, and IL-4 gene were determined by RT-qPCR. As geometric mean ± SD (three DLNs). The relative quantification was performed by the comparative Ct method (∆∆Ct), using the lymph node from the control group as a calibrator (fold change = 1). ^∗^*P* < 0.05, significant decrease or increase in cytokine gene expression in the vaccinated group draining lymph nodes compared to the control group.

The gene expression of interleukin-12 (IL-12), interferon gamma (IFN-*γ*), and interleukin-4 (IL-4) was assessed in the draining lymph nodes (DLNs) of the vaccinated group before challenge and 2, 4, and 6 weeks after challenge in order to determine whether the cellular immune response induced by vaccination is associated with a cytokine expression. During immunization, the gene expression of IFN-*γ*, IL-4, or IL-12 in the draining lymph nodes (DLNs) of vaccinated mice groups had no differences compared with their gene expression in the draining lymph nodes (DLNs) of the control group with *P* > 0.05. These study results showed an increase in the gene expression of IFN-*γ* in draining lymph nodes (DLNs) of mice vaccinated with the pCI-L5 vaccine at weeks 2, 4, and 6 (4.29-fold (*P* < 0.05), 8.1-fold (*P* < 0.05), and 19.6-fold (*P* < 0.05), respectively), compared with the gene expression of IFN-*γ* in draining lymph nodes (DLNs) of the control group at the same weeks (Figure 7(a)). Also, these study results showed an increase in the gene expression of IL-12 in draining lymph nodes (DLNs) of mice vaccinated with the pCI-L5 vaccine at weeks 2, 4, and 6 (10-fold (*P* < 0.05), 13.75-fold (*P* < 0.05), and 21-fold (*P* < 0.05), respectively), compared with the gene expression of IL-12 in draining lymph nodes (DLNs) of the control group at the same weeks (Figure 7(b)). Finally, these study results showed a decrease in the gene expression of IL-4 in draining lymph nodes (DLNs) of mice vaccinated with the pCI-L5 vaccine at weeks 2, 4, and 6 (0.52-fold (*P* < 0.05), 0.57-fold (*P* < 0.05), and 0.54-fold (*P* < 0.05), respectively), compared with the gene expression of IL-4 in draining lymph nodes (DLNs) of the control group at the same weeks (Figure 7(c)).

The calculation of the ratio of the gene expression of IFN-*γ* to the gene expression of IL-4 (IFN-*γ*/IL-4 ratio) in the draining lymph nodes (DLNs) of the study and control groups before challenge and 2, 4, and 6 weeks after challenge was made as an indicator of potential immunization.

**Figure 8:**
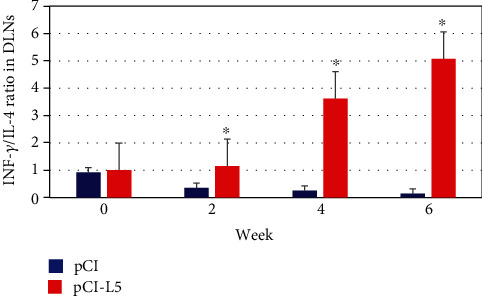
IFN-*γ*/IL-4 ratio in draining lymph nodes (DLNs) obtained from BALB/c mice inoculated with 100 *μ*g pCI-L5 vaccine before and after challenge with *L. tropica*. The IFN-*γ*/IL-4 ratio in response to pCI-L5 and pCI is compared (^∗^*P* < 0.05).

Before challenge, the IFN-*γ*/IL-4 ratio of the vaccinated mice group had no differences compared with the ratio of the control group with *P* > 0.05. The IFN-*γ*/IL-4 ratio of the vaccinated mice group was higher than the IFN-*γ*/IL-4 ratio of the control group at weeks 2, 4, and 6 postchallenge with *P* < 0.05 (Figure 8).

## 4. Discussion

Cutaneous leishmaniasis (CL) in anthroponotic form has been present long ago throughout the history of Syria. *Leishmania tropica* is the causative agent of this form. After the deterioration of the health situation in Syria due to the war, a significant increase in the number and distribution of CL cases across the country has been registered as discussed by Muhjazi et al. [20]. Treatment of cutaneous *leishmania* is currently based on drugs which are toxic and have severe side effects. Also, resistance to these drugs have developed, so new antileishmania drugs or a vaccine is much more important as discussed by Ghaffarifar et al. [21].

The search for an effective vaccine is an important goal for researchers generally and Syrians especially. DNA vaccination represents an important choice as an effective vaccine against cutaneous leishmaniasis since it activates a desirable and specific TH1 cellular response which is needed to face cutaneous leishmaniasis as discussed by Carrión et al. [22].

In order to use genes as a DNA vaccine against *Leishmania tropica* infection, we need to know information about the existence of these genes in the *Leishmania tropica* genome and their essential roles in the life of the parasite and their potential immunogenicity. Ribosomal protein L5 attaches to 5 S rRNA ensuring intracellular transport of 5 S rRNA. This event plays a vital role in ribosome assembly; thus, ribosomal protein L5 has a fundamental role in the protein synthesis process as discussed by Matthew and Dreyfuss [15]. Also, ribosomal protein L5 as member of the ribosomal protein family is considered a pan antigen which provides the immunomodulatory properties needed for vaccine design as discussed by Requena et al. [8] and Santarém et al. [9].

This study chose the ribosomal protein L5 gene to design a DNA vaccine against the infection with *L. tropica* which is the causative agent of major cutaneous cases in Syria. The presence of the ribosomal protein L5 gene was not demonstrated in *L. tropica*, so this systematic study was first made to investigate if the ribosomal protein L5 gene existed in the *Leishmania* Syrian strain genome, to prove if there is an expression of the L5 gene in these parasites, and to sequence this gene. Then, the ribosomal protein L5 gene was inserted into the pCI plasmid, resulting in the pCI-L5 DNA vaccine.

BALB/c mice were immunized by the designed vaccine. The cellular immunity response elicited by the DNA vaccine in immunized BALB/c mice was studied and compared to the control group which was inoculated with an empty plasmid (pCI). Lesion development was determined and measured for 6 weeks after the challenge. At the last week after the challenge, the parasite burden has been determined in the footpad lesion and local draining lymph nodes. RT-qPCR was used to quantify the gene expression of the cytokines (IFN-*γ*, IL-12, and IL-4) in the draining lymph nodes (DLNs) of BALB/c mice.

In the study group, T helper 1 (TH1) cytokines like IFN-*γ* and IL-12 are the crucial factors in the initiation of protective immunity against *leishmania* infection, whereas T helper 2 cytokines (TH2) like IL-4 facilitate the persistence of parasites by downregulating the TH1 immune response as discussed by Maspi et al. [23]. The cellular immune responses in leishmaniasis have been extensively studied in mouse models. Susceptible mice (BALB/c) develop progressive lesions, with a predominance of the TH2 response, leading to the production of anti-inflammatory cytokines, such as IL-4. Resistant mice display small lesions with few parasites and a predominance of IFN-*γ* and IL-2 cytokines, characteristic of a TH1 response. These latter cytokines activate leishmanicidal mechanisms in infected macrophages, with high ROS and NO production, leading to parasite killing as discussed by Santos and Brodskyn [24].

The presence of parasites at the end of the challenge at the footpad lesion and local draining lymph nodes may be due to the presence of IL-10 which is an important regulatory cytokine involved in parasite persistence in mice, and their major source is CD4+CD25+ regulatory T cells as discussed by Stober et al. [25].

In the control group, the concentration of IL-4 increases after leishmaniasis that results in aggravation of the infection as discussed by Sacks and Trauth [26]. IL-4 plays an important role in the differentiation of TH0 cells into TH2 cells. IL-4 is mainly produced by TH2, and its production drives inhibition of leishmanicidal activity of macrophages and prolonged survival of parasites. IL-4 limits the generation of TH1 cytokines through downregulating IL-12 production. Furthermore, IL-4 downregulates the production of chemokines that recruit TH1-type cells to the infection site as discussed by Maspi et al. [23].

The correlation between a polarized immune response and outcome of infection had led to the fact that the predominance of TH1 or TH2 responses determines the outcome as discussed by Teshager and Mohammed [27]. So, the efficacy of a studied vaccine requires determination of the type of immune response as discussed by Tripathi et al., i.e., whether it is a TH1 or TH2 response [28].

Some studies demonstrated that certain strains of *L. tropica* cause mild pathogenesis in BALB/c mice characterized by small or no detectable swelling in the injection site, so measuring lesion size was not considered as a promising tool for the evaluation of vaccine efficiency as discussed by Fatemeh et al. [17] and Rostamian et al. [29]. In fact, in some cases, cutaneous lesions due to *L. tropica* may develop to ulcerative detectable lesions as discussed by Masoudzadeh et al. [30], like the case in our study, so monitoring lesion size could strongly reflect the immune response obtained from the vaccines used.

The pCI-L5 vaccine was able to induce partial protection against infection with *Leishmania tropica*, and the ratio of IFN-*γ*/IL-4 in the draining lymph nodes (DLNs) of mice of the study group was 2.83 and 3.62 at the second and fourth weeks after the challenge, respectively, and then it stabilized at the value of 5.07 at the sixth week (greater than 1). Therefore, the cell-mediated immune response caused by the vaccine after two weeks was the TH1 type since the ratio of IFN-*γ*/IL-4 was greater than 1, and this ratio was sufficient to induce partial immunity against *Leishmania tropica*, which is associated with a decrease in the size of the lesion as discussed by Masoudzadeh et al. [30] and the survival of fewer parasites in the lesions and in the draining lymph nodes after the sixth week of the challenge.

This indicates the importance of the ribosomal protein L5 gene and the possibility of using it in subsequent experiments with an adjuvant or protein encoded by the same gene, allowing for increased immunization efficacy as discussed by Todolí et al. [31], or using the ribosomal protein L5 gene as part of a combined vaccine as obtained in other studies, which showed a higher intensity of prophylactic immunity when using a mixture of recombinant plasmids compared to immunization with each recombinant plasmid separately as discussed by Rafati et al. [32].

Studies also indicate that increasing the dose is necessary to obtain better results, indicating the possibility of increasing the dose in our study to get better results. We could not compare our results because there are no similar studies that use the ribosomal protein L5 gene as a DNA vaccine against Leishmaniasis caused by *Leishmania tropica.*

In this study, the DNA vaccine immune response is only due to the effect of the recombinant plasmid with the ribosomal protein L5 gene, because the pCI plasmid alone is unable to induce an immune response against leishmaniasis since it does not contain CPG motifs, while the pTCAE plasmid alone was able to induce partial immune response in mice against *L. major* because the pTCAE plasmid contains CPG motifs as discussed by Vakili et al. [33].

According to these data, the ribosomal protein L5 gene is a part of the *L. tropica* genome, and the ribosomal protein L5 is expressed in the *L. tropica* parasite. It is suggested that the immune response of mice vaccinated with the pCI-L5 vaccine is the TH1 response; nevertheless, this response was insufficient and the addition of an adjuvant or protein encoded by the same gene or an increased dose is needed to elevate this response. This activation leads activated T cells to produce IFN-*γ*, which in turn induces macrophages to secrete IL-12, kill the parasites, and control the infection.

## 5. Conclusion

This study has demonstrated that the ribosomal protein L5 gene is a part of the *L. tropica* genome, and the ribosomal protein L5 is expressed in the *L. tropica* parasite. For the first time, we proved the presence of the ribosomal protein L5 gene in *L. tropica*, defined the sequence of the cDNA of the ribosomal protein L5 gene, and submitted this gene to Genbank with accession number MF495877.1. The data reported in this study suggest that there was a decrease in the dermal lesion size in BALB/c mice immunized with ribosomal protein L5 as a DNA vaccine, but the parasite still survived at the inoculation site and in the draining lymph nodes (DLNs), and the ratio of IFN-*γ* to IL-4 was 5.07 with the pCI-L5 vaccine. According to these data, the immune response type in mice vaccinated with this vaccine was TH1, but it is still insufficient, and the addition of an adjuvant or protein encoded by the same gene or an increased dose is needed to elevate this response and improve the vaccine's performance to protect against further infections that might occur in endemic areas.

## Data Availability

All data created or used during this study are openly available from the Leishmania Centre for Epidemiological and Biological Studies, Damascus University.

## Supplementary Materials

Supplementary Materials Nucleotide sequence of L5 cDNA for *L. donovani*, *L. infantum*, *L. major*, *L. mexicana*, and *L. tropica*. The deduced amino acid sequence is indicated below the cDNA sequence.
