# Supplementary material for: Investigating the Existence of Ribosomal Protein L5 Gene in Syrian Strain of Leishmania tropica Genome: Sequencing It and Evaluating Its Immune Response as DNA Vaccine
Source: J Parasitol Res. 2021 May 20;2021:6617270. doi: 10.1155/2021/6617270 (PMC8163552; doi:10.1155/2021/6617270)

**Supplementary Material. Nucleotide Sequence of L5 cDNA for *L.donovani , L.infantum , L.major, L.mexicana,* and *L.tropica*. The deduced amino acid sequence is indicated below the cDNA sequence.**


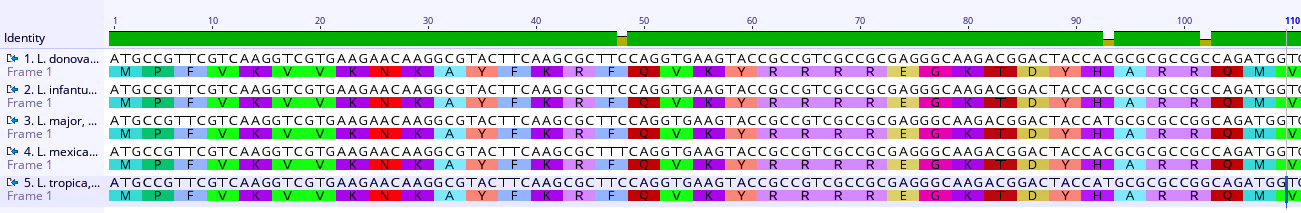


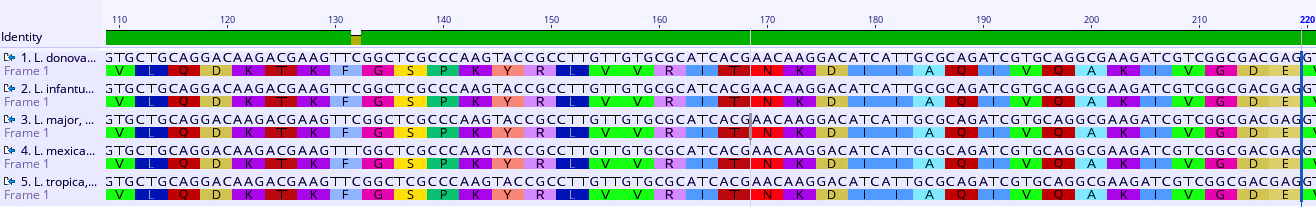


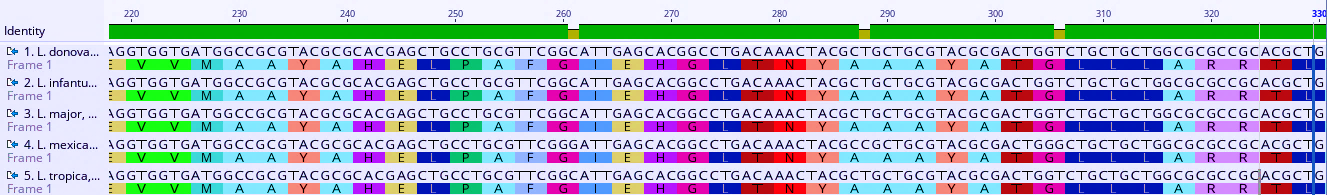


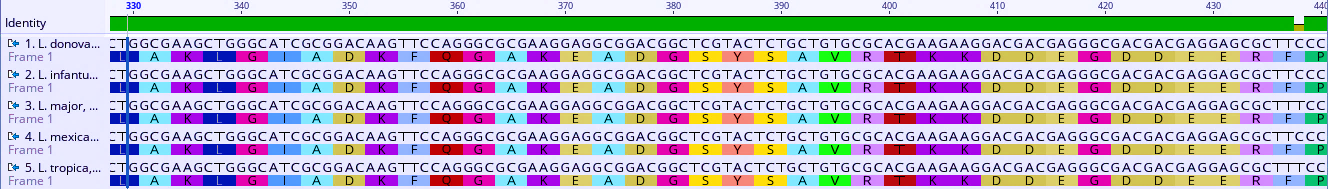


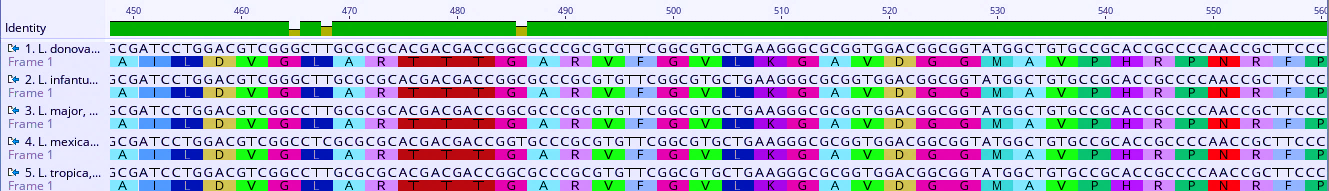


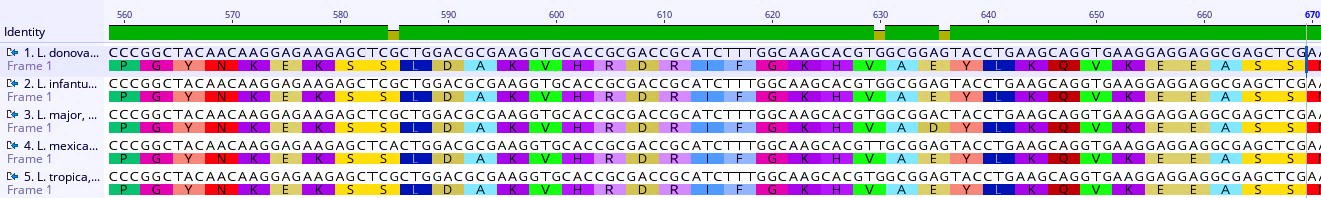


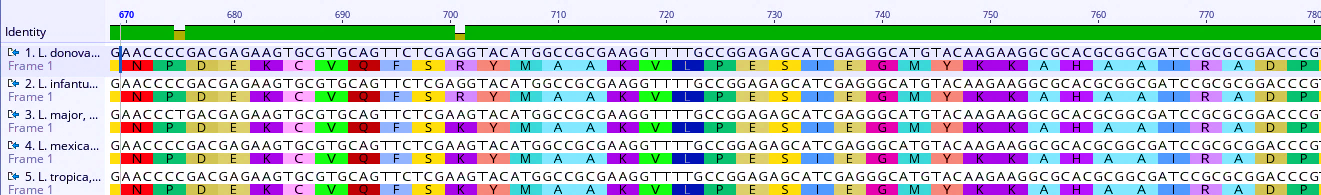


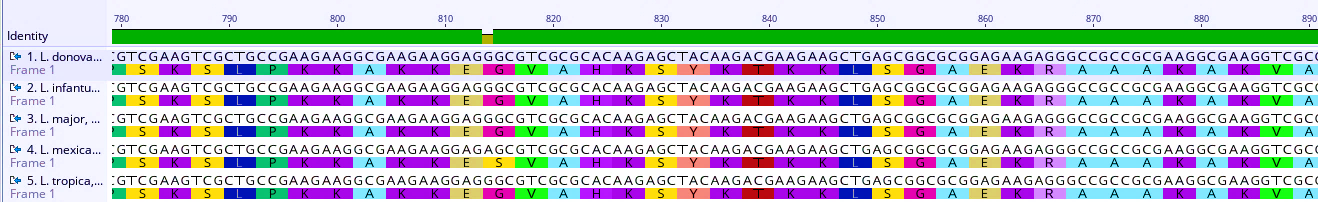


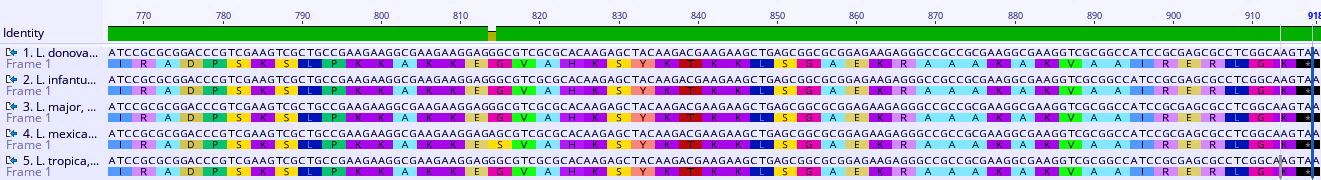

Supplement: Supplementary Materials — Nucleotide sequence of L5 cDNA for L. donovani, L. infantum, L. major, L. mexicana, and L. tropica. The deduced amino acid sequence is indicated below the cDNA sequence. [file 6617270.f1.docx]
